# Improvement of Dielectric Breakdown Performance by Surface Modification in Polyethylene/TiO_2_ Nanocomposites

**DOI:** 10.3390/ma12203346

**Published:** 2019-10-14

**Authors:** Weiwang Wang, Shengtao Li

**Affiliations:** State key laboratory of electrical insulation and power equipment, Xi’an Jiaotong University, Xi’an 710049, China; stli@mail.xjtu.edu.cn

**Keywords:** nanocomposites, surface modification, traps, interface, breakdown

## Abstract

Dielectric breakdown is a significant property for the insulation system in high voltage power equipment. This paper is dedicated to the improvement of dielectric breakdown by surface-functionalized nanoparticles in low-density polyethylene (LDPE). Prior to the preparation of LDPE/TiO_2_ nanocomposites, the nanoparticles were surface modified by the silane coupling followed by the chemical reaction process. Results of Fourier transform infrared spectroscopy (FTIR) indicated that some polar groups and chemical bonding were introduced on the surface of TiO_2_ nanoparticles. A reduction of dielectric permittivity was observed at low nanoparticle loading (<2 wt%) samples, which responded to the restriction of the molecular chain in the interface region. High nanoparticle loadings (2 wt%, 5 wt%, 10 wt%) introduced an obvious relaxation polarization. The trap parameters detected by the thermally stimulated current (TSC) method indicated that the deep traps were introduced by small amounts of nanoparticles (≤2 wt%), while more shallow traps occurred in high loading (5 wt%, 10 wt%) samples. Meanwhile, the increase of breakdown strength at low loading samples were closely related to the deep traps, which was ascribed to the interface region by surface chemical modification.

## 1. Introduction

The incorporation of inorganic nanoparticles into the polymer matrix opens a new pathway for engineering flexible composites that exhibit advantageous electrical [1], mechanical, and thermal properties [2]. Polymer nanocomposites with a well-dispersed homogeneous blend present favorable electrical properties, such as decreasing dielectric permittivity, improving electric strength, restraining space charge accumulation, and improving partial discharge (PD) resistance [3]. Previous work demonstrated that the interface is recognized as the dominant factor of dielectrics at the nanometric level, and the interfacial region is described outside of the nanoparticle surface in terms of an extended Helmholtz double layer or Gouy–Chapman layer [4].

The dielectric breakdown performance has been studied in polyethylene nanocomposites with various nanofillers, especially for the surface chemical modifications. Polyethylene/montmorillonite (MMT) clay nanocomposites exhibit reduced breakdown strength with the poorly dispersed MMT nanofillers, while the increase of breakdown strength occurred in the good dispersion nanocomposites with much more irregular lamella structures (an increase in the *α* value from ~120 to ~180 kV/mm) and an increase in *β* from ~10 to ~20) [5]. Previous papers have been focused on the breakdown strength in low-density polyethylene (LDPE) nanocomposites [6,7,8]. The results pointed out that slight nanofillers (<2 wt%) are beneficial to the increase of breakdown strength, while high contents of nanofillers (>2 wt%) reduce the breakdown strength [7,8]. The DC breakdown voltage endurance of cross-linked polyethylene (XLPE)/silica (SiO_2_) nanocomposites indicate an increase in breakdown strength for the untreated nano-filled composites [9]. The XLPE/SiO_2_ nanocomposites present a time to failure which is two orders of magnitude higher than that of base resin [9]. Particularly, the different surface modifications of nano-silica could enhance the breakdown strength than that of untreated nanofillers. For example, the aminopropyl-trimethoxysilane (AEAPS) and vinylsilane (TES) treated nano-silica fillers present higher breakdown strengths (over 400 kV/mm) than that of untreated nanofillers (314 kV/mm) [9]. In another report, the addition of untreated TiO_2_ nanofillers (5 wt%) decrease the DC breakdown strength. However, the AEAPS treated TiO_2_ nanofillers (5 wt%) enhance the DC breakdown strength [10].

Nanoparticles, acting as a sort of hindrance to the treeing mechanism, are capable of improving dielectric strength in nanocomposites. The interparticle distances are quite small, therefore the volume of a polymer that is devoid of particles is reduced and the nanoparticles act like barriers to the flow of electric current between the electrodes [11]. However, in comparison with the micro composites, particularly the surface-modified nanoparticles, the roles of the interface between the nanoparticle and polymeric matrix are detrimental to the dielectric breakdown [12,13,14]. Rather low content of nanofillers can manipulate the dielectric breakdown strength by tailoring the surface of the nanoparticles, and hence, the interfacial region [10]. The proposed multi-core model indicates that the strong bonded region and relaxation region could hinder the charge transport and electron acceleration [15]. The potential barrier model proposes a multi-function area around the nanoparticle [16]. The high and low levels of trap barriers are introduced at the interface, resulting in the enhancement of the dielectric strength [16].

It indicates that the polymer morphology, especially for the interface can be modified by surface chemical bonding, crystallization, and chain conformation, indicating the formation of the traps [17]. Deep traps are created by nanoparticles [8,9]. In the case of surface modification, the number of deep trap sites is introduced by surface polar chemical groups in XLPE/silica nanocomposites [9]. In addition, the deep traps are also introduced in polyimide/silica nanocomposites [18]. It is postulated that the addition of new traps can connect to the original traps, or replace the original traps [15]. Previous work pointed out that the formed barrier traps at the interface interact with the original traps (or overlap), leading to an increase of deep traps [8]. It is generally accepted that the appropriate deep traps are in favor of the improvement of breakdown performance by reducing the charge carrier mobility and energy accumulation [9,17,19]. 

In order to study further the link between the surface modification of nanoparticles and the dielectric breakdown, this paper focused on the effects of surface chemical treatment of TiO_2_ on the dielectric breakdown of LDPE nanocomposites. The surface-modified effects, such as the chemical groups, dielectric response were revealed by SEM, FTIR, and dielectric spectra. After that, the traps were studied by the thermally stimulated current (TSC) method. The improvement of the breakdown strength of LDPE/TiO_2_ nanocomposites is discussed followed by the relationship between trap formation and surface modification.

## 2. Materials and Experimental Methods 

The polymer matrix used in this work was LDPE (DowDuPont 681I) [20]. TiO_2_ nanoparticles, rutile phase, 30 nm average diameter from Hangzhou Wanjing Company (Hangzhou, Zhejiang, China) were used. The surface area of the TiO_2_ nanoparticle was about 150 m^2^/g, and the relative permittivity of the fillers was about 173 (*ε*_r_). 3-glycidoxypropyl-trimethoxy-silane (KH560) was selected as a coupling agent for surface modification.

Before modification, the nanoparticles were dried under 120 °C for 12 h. The surface modification of TiO_2_ nanoparticles was carried out by the toluene reflux method. Firstly, the dried nanoparticles were dispersed into the absolute ethyl alcohol and treated by a high-pressure homogenizer to reduce the agglomeration. Then the absolute ethyl alcohol was evaporated at 60 °C for 10 h. Secondly, 1 g nanoparticles were mixed with 60 mL toluene in a three-necked, round-bottomed flask, forming the suspension. The suspension was treated using stirring and ultrasonic dispersing. Thirdly, the treated suspension (stirring) was mixed with 6 mL KH560 and chemically reacted under 110 °C in a backflow device. The KH560 was added into the suspension by three stages, and the duration of the chemical reaction was 24 h. Fourthly, the suspension was centrifuged under 3000 r/min for 5 min. This process was repeated several times for removing the residual toluene and unreacted KH560. Finally, the surface-modified nanoparticles were dried at 120 °C and ground for material preparation.

LDPE/TiO_2_ nanocomposites were prepared by melt blending method, which was described in previous work [8]. The different contents of TiO_2_ nanoparticles were added into the LDPE matrix, including 0.1 wt%, 0.5 wt%, 1 wt%, 2 wt%, 5 wt%, and 10 wt% weight percentage nanoparticles. The samples about 0.2 mm thickness were prepared by hot-pressing for the measurements.

**Figure 1:**
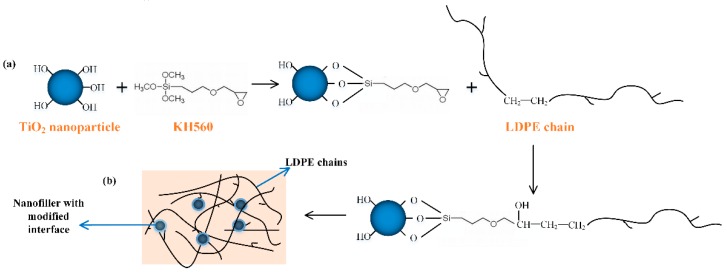
Schematic of surface modification and nanocomposite. (**a**) Surface modification of TiO_2_ nanoparticle; (**b**) schematic figure of low-density polyethylene (LDPE)/TiO_2_ nanocomposites matrix.

Figure 1 shows the flowchart of the surface modification. The (OCH_3_)_3_–Si– groups were reacted with the surface hydroxyl of TiO_2_ nanoparticles, and the alkoxy was linked to the LDPE chain. Consequently, the TiO_2_ nanoparticle surface could be modified, as shown in Figure 1a. Similar treatment can be found in Reference [21]. Figure 1b shows the schematic diagram of the dispersed TiO_2_ nanoparticles with interfaces in the LDPE matrix. After surface modification, an interfacial region around the nanoparticle is introduced into the matrix. 

**Figure 2:**
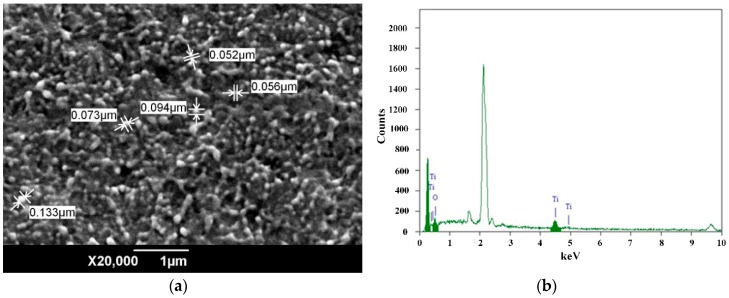

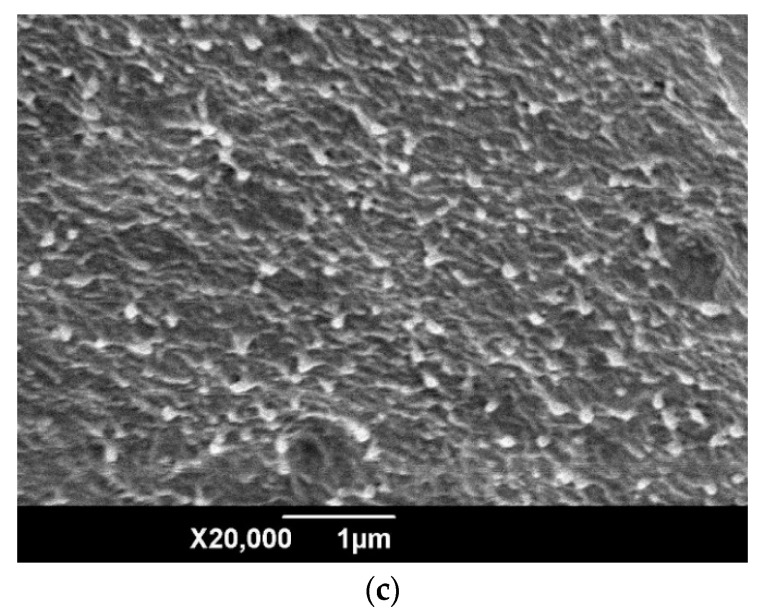
SEM images and Energy Dispersion Spectrum (EDS) analysis of LDPE/TiO_2_ nanocomposite; (**a**) 1 wt% sample; (**b**) EDS of the 1 wt% sample; (**c**) SEM image of 5 wt% sample.

Figure 2 shows the scanning electron microscope (SEM) result of the 1 wt% and 5 wt% samples (cross-section). The images show the good dispersion of the nanoparticles. The majority of the nanoparticles were dispersed uniformly with a diameter of less than 100 nm. Furthermore, the result of the Energy Dispersion Spectrum (EDS) spectrum of 1 wt% sample indicates the existence of Ti and O elements in the TiO_2_ nanoparticles, as shown in Figure 2b. It can be verified that the TiO_2_ nanoparticles were introduced into the LDPE matrix. 

A wide-band dielectric spectrometer (Novocontrol Concept-80, Germany) was used to measure the permittivity and dielectric loss of the LDPE/TiO_2_ nanocomposites. Two sides of the sample surface were sputtered by gold electrodes with a diameter of 30 mm before the measurement. The measurements were carried out under room temperature. The dielectric parameters were measured in the frequency range of 10^−2^ Hz to 10^6^ Hz. Three samples were used for each kind of nanocomposites to verify the repeatability of the experiments. One of the results was selected in this paper.

Electric breakdown strengths of all specimens were measured in terms of the previous work [7]. The ramp rate of 0.5 kV/s of DC voltage was used. Fifteen breakdown points of each loading sample were tested for breakdown analysis. The mean value and deviation of the calculated Weibull parameters were discussed.

Trap information of the LDPE/TiO_2_ nanocomposites were analyzed by thermally stimulated depolarization current (TSDC) measurement. The depolarized currents were obtained with the temperature range of −100 °C to 80 °C, which is described in the previous paper [8]. TSDC curves of each loading sample were tested five times. The mean value and deviation of the calculated trap parameters were analyzed.

## 3. Experimental Results 

### 3.1. FTIR Detection

**Figure 3:**
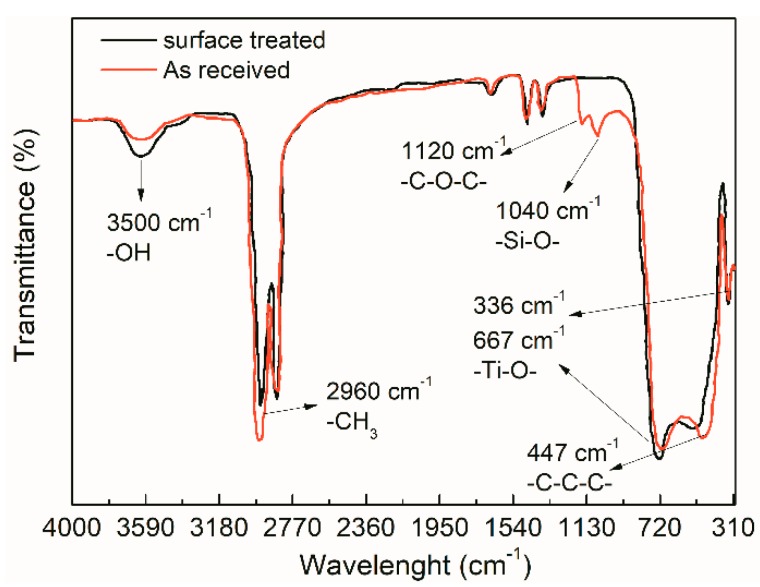
FTIR results of as received and modified TiO_2_ nanoparticles.

The FTIR result of as-received and surface-modified TiO_2_ nanoparticles was measured by FTIR device, IRPrestige-21, FTIR-8400S. Figure 3 shows the transmittance peaks of the FTIR result with the wavelength. It indicates that the new peaks occurred at 1120 cm^−1^ and 1040 cm^−1^, respectively. These stretching vibration absorption peaks present the –C–O–C– (1120 cm^−1^) and Si–O– (1040 cm^−1^) chemical bonds, which were ascribed to the chemical reaction between the coupling agent and the surface hydroxyl of TiO_2_ nanoparticles. In addition, compared to the as-received TiO_2_ nanoparticles, the absorption peak of hydroxyl (–OH) at 3500 cm^−1^ decreased in the surface-modified nanoparticles. It is accepted that the surface hydroxyl (–OH) of the TiO_2_ nanoparticles reduces by the chemical reaction with the coupling agent molecules. An increase of group –CH_3_ peak at 2960 cm^−1^ showed an increase after modification, which was generated by the coupling agent molecules. Some small molecule groups existed at a wavelength of less than 1000 cm^−1^. These peaks presented the Ti–O, –CH, –CO, –CN groups, indicating a slight increase after the surface modification. Hence, it can be concluded that the surface modification introduced the chemical bonding and polar groups on the TiO_2_ nanoparticle surface. 

In view of the FTIR analysis of surface-modified TiO_2_ nanoparticles, we could draw the conclusion that strong chemical bonding and interaction occurred between the nanoparticle and the LDPE matrix (at the interface). Molecules of the coupling agent were grafted on the nanoparticle surface and linked to the LDPE chains. Together with the surface effect, the quantum size effect of nanoparticle, the dielectric performance of the LDPE/TiO_2_ nanocomposites could be improved. 

### 3.2. Dielectric Permittivity

**Figure 4:**
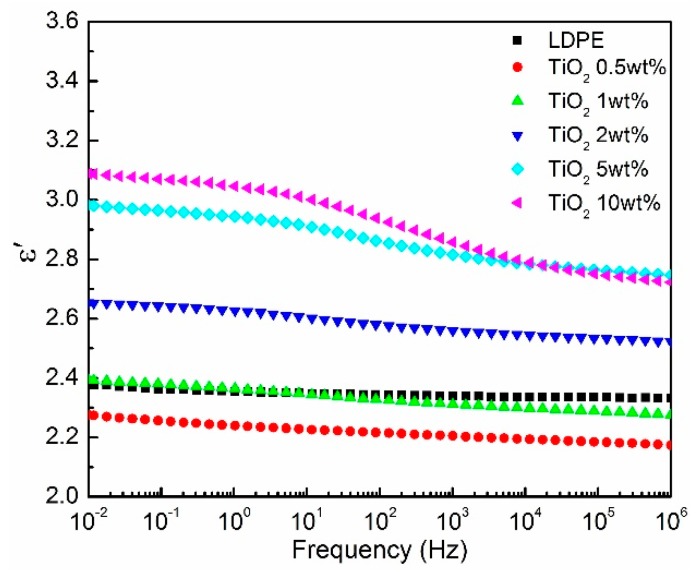
Frequency dependence of real part permittivity of LDPE/TiO_2_ nanocomposites.

Figure 4 shows the real part permittivity of the LDPE/TiO_2_ nanocomposites. It indicated that the permittivity of the neat LDPE matrix showed a stable value (*ε*_r_ ~2.36) in the frequency range due to its nonpolar dielectric. However, the used LDPE polymer usually contains some defects and additives during the manufacture, such as antioxygen and stabilizer, forming some polar groups. Consequently, the permittivity presented a slight decrease in the high-frequency region, as shown in Figure 4. Interestingly, the permittivity showed a reduction at the low loading samples (<2 wt%). Furthermore, the decrease of permittivity with the frequency became obvious, indicating more polar groups were introduced in the nanocomposites. At the high loading (>2 wt%), an obvious increase of permittivity (higher than that of neat LDPE) could be obtained, as shown in Figure 4. In addition, high permittivity occurred at low frequency, while low permittivity existed at high frequency. This could be responsible for the Debye polarization theory [22].

The permittivity characteristics of LDPE/TiO_2_ nanocomposites agreed with the previous works [1,7,23]. It has been postulated that the tethered entanglement associated with nanocomposites could reduce the free volume in the polymer [24], leading to a decrease of permittivity. However, measurements of free volume in polymer nanocomposites demonstrate that the free volume increases or presents little change [24,25]. Most importantly, the reduction of permittivity is closely related to the chain conformation and configuration at the interface region. A short-range highly immobilized layer develops at the interface, resulting in the restriction of the chain movements [1]. A weak polarization region with a large interface thickness contributes to the reduction of permittivity [23]. The chain morphology at the interface region was closely related to surface modification. The nanoparticles played dominant roles in dielectric response at high loading samples (>1 wt%). The agglomeration and the strong interaction between fillers contributed to the excepted Maxwell–Wagner interfacial polarization [24], corresponding to the formation of the relaxation processes.

### 3.3. TSDC Results

#### 3.3.1. Relaxation Process from TSDC Results

**Figure 5:**
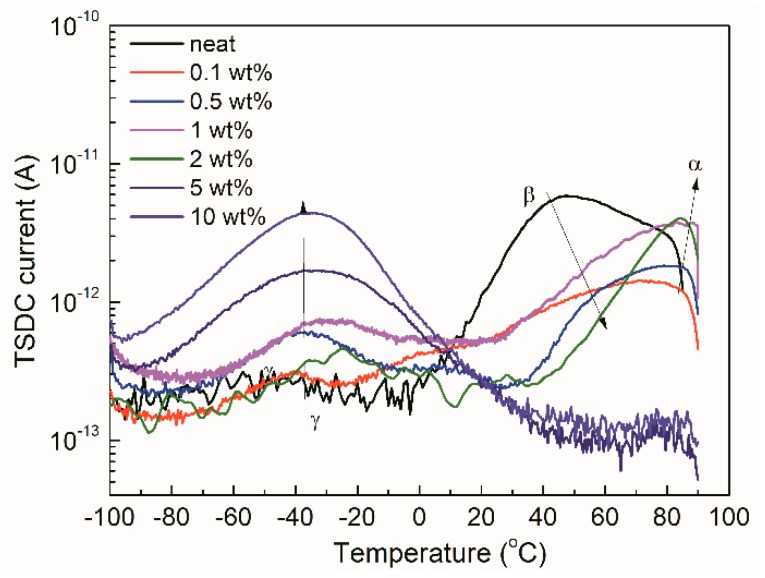
Thermally stimulated depolarization current (TSDC) results of neat LDPE and LDPE/TiO_2_ nanocomposites.

As the previous study has shown, a TSDC plot of polyethylene exhibits three relaxation processes, including *γ*, *β*, and *α* relaxation processes [26], as shown in Figure 5. *γ* relaxation is probably determined by the movements of side groups and chain segments in the amorphous region. It is likely caused by the defects in the amorphous region (surface traps). *β* relaxation is derived from the dipolar polarization. Some interface defects and shallow traps associated with this process can be determined. The release of space charge followed by trapping of charge carriers in deep traps contributes to the α process. 

Figure 5 shows the TSDC plots of LDPE/TiO_2_ nanocomposites. Incorporating with the TiO_2_ nanoparticles altered the value and position of relaxation peaks in the TSDC results. At low loadings (≤2 wt%), the α peak increased and shifted to high temperatures, the *β* peak decreased and moved to high temperature, while the *γ* relaxation presented a slight increase with the loading. It is worth noting that the *γ* relaxation presented a significant increase at a high loading sample (>1 wt%). It shifted slightly to the high temperatures with the increase of loading. Furthermore, the α peak becomes much smaller for the 5 wt% and 10 wt% samples. Similar TSDC results are described in LDPE/Al_2_O_3_ nanocomposites [8]. 

**Figure 6:**
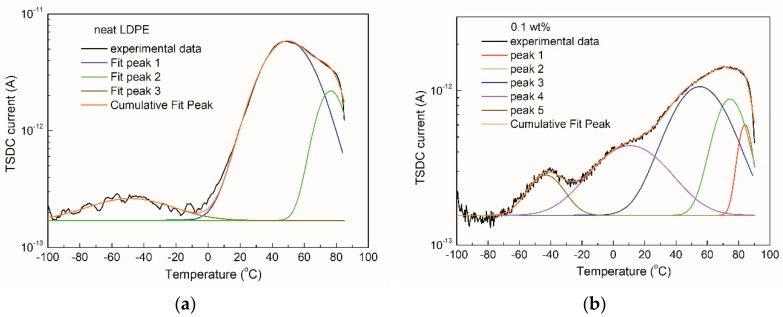
Fitting results of LDPE/TiO_2_ nanocomposites, indicating the multi-relaxation processes in the nanocomposites. (**a**) Neat LDPE; (**b**) 0.1 wt%.

In order to evaluate the trap parameters underlying the TSDC results, it is necessary to fit the relaxation processes of the TSDC plots. Figure 6 shows examples of the fitting curves in the TSDC results. Three typical peaks, that is α, *β* and *γ* peaks could be well fitted for the neat LDPE. In the case of 0.1 wt% sample, five peaks were used to fit the TSDC plot. Among them, peak 1 and 2 associated with the *α* relaxation presented the deep traps. The peaks 3 and 4 (*β* relaxation) indicated the shallow traps. The peak 5 (*γ* relaxation) was a kind of much shallower trap related to the charge carrier mobility.

#### 3.3.2. Trap Parameters 

Table 1 shows the calculated trap level and amount of trapped charges according to the “initial rise method” [8]. It indicated that LDPE showed three kinds of traps with the trap depth of 1.1 eV, 0.6 eV, and 0.06 eV, respectively. The traps of 0.6 eV (*β* relaxation) with the 6.6 nC played the dominant role. In the case of low loading samples (≤2 wt%), it is interesting that the new deep traps were introduced compared with the LDPE. The introduced new relaxation peaks occurred at nearly 85 °C, indicating a trap depth of over 1.2 eV. In the case of original deep traps (peak 2), its trap depth slightly decreased. Peak 3 revealed the shallow traps (*β* relaxation), indicating little change in the trap depth, while an obvious reduction of trap density occurred with loading. Only shallow traps existed at high loading samples (5 wt% and 10 wt%), as shown in Table 1. A kind of shallower traps was calculated (~0.1 eV) from peak 5. It showed an increase in loading and played a dominant role in high loading samples.

**Figure 7:**
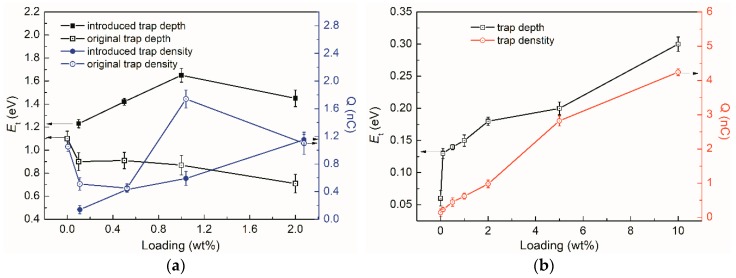
Trap information of LDPE/TiO_2_ nanocomposites. (**a**) Deep traps; (**b**) shallow traps.

Figure 7 shows the influence of nanoparticle loading on both deep and shallow traps parameters (data from Table 1). For the low loading samples (≤2 wt%), the introduced new deep trap depth and density showed an increase with loading. In addition, the original deep trap depth slightly decreased (1.1 eV→0.72 eV). Its trap density exhibited an initial decrease and then increase with loading, as shown in Figure 7a. The decrease of original traps was probably derived from the strongly bound polymer chains at the interface region. However, the increase of the traps was likely due to the interaction between nanoparticles (1 wt% and 2 wt%). Figure 7b shows the increase of shallow traps with the loading. It appeared that the trap depth and density were quite small at low loadings (<2 wt%). However, it significant increased at high loadings (>2 wt%). It turned out that the large amounts of TiO_2_ nanoparticles contributed to the shallow traps in the nanocomposites.

### 3.4. Dielectric Breakdown

**Figure 8:**
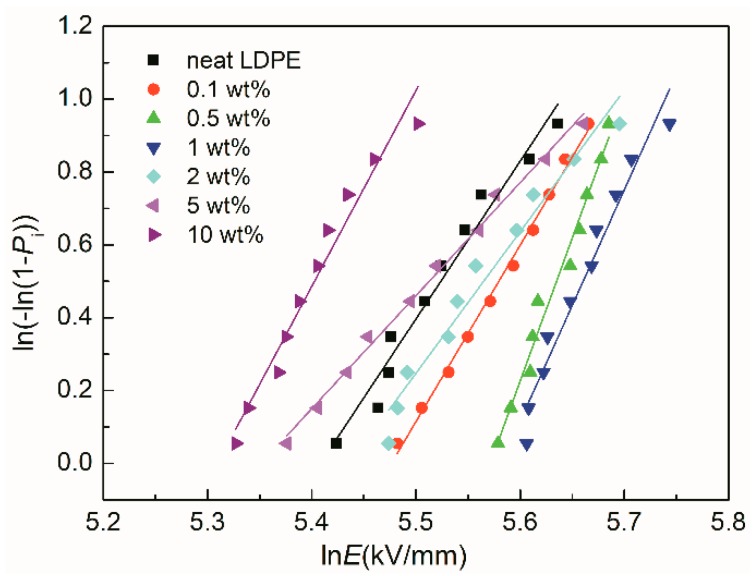
Breakdown results of LDPE/TiO_2_ nanocomposites using the Weibull distribution method.

A typical two-parameter Weibull distribution method [27] was employed to analyze the breakdown data of LDPE/TiO_2_ nanocomposites. A straight line was obtained by Weibull analysis, as shown in Figure 8. The position of the fitted line (ln*E* direction) presents the breakdown strength, and the slope of the line indicates the dispersibility of the breakdown data.

The shape parameters *α*′ (breakdown strength) and *β*′ can be calculated by fitting the slope of the data in Figure 8. Table 2 shows the calculated scale and shape parameters. *α*′ (breakdown strength) increased initially and then decreased with the loading. The maximum breakdown strength occurred at 1 wt% sample, which was enhanced by 19% compared to that of neat LDPE. However, the high loading samples exhibited low breakdown strength. This phenomenon agrees with the previous work [8]. In the case of the shape parameter *β′*, incorporating with slight TiO_2_ nanoparticles (<2 wt%) increased *β′*. It indicated that the data dispersion of the breakdown was improved by TiO_2_ nanoparticles (<2 wt%). However, it decreased at high loading samples (2 wt%, 5 wt%). This was probably ascribed to the effects of nanoparticles (agglomeration).

## 4. Discussion

It is generally accepted that the traps play significant roles in the dielectric breakdown of nanocomposites [18,28,29]. The increase of breakdown strength was explained in terms of the trapping effect by the fillers, which prevented electron acceleration [30,31]. The decrease of breakdown strength in nanocomposites is mainly ascribed to the interaction between nanoparticles, or the percolation effect [32]. In this paper, the improvement of the dielectric breakdown of LDPE by slight TiO_2_ nanoparticles is mainly discussed. 

It has been established that modification of traps, especially for deep traps, played dominant roles in the improvement of dielectric breakdown of LDPE/TiO_2_ nanocomposites. TiO_2_ nanoparticles, due to the surface modification, the extended surface area, and the conformation of LDPE chains were modified. The trap depth and density were altered by the effects of the interface region, leading to the reduction of the charge carrier mobility and the accumulated energy. Deep traps were introduced by the small amount of TiO_2_ nanoparticles, as shown in Figure 7. Deep traps can capture electrons and suppress the formation of “hot” electrons [33], leading to the reduction of low-density regions and improve the breakdown properties. In addition, deep traps are beneficial to improve space charge performance. The injected electron and hole charges at the electrode/sample interface were easily captured by the deep traps, resulting in the formation of homo-charges accumulation. Consequently, the charge injections were restricted, indicating a reduction of the charge carrier for DC breakdown.

**Figure 9:**
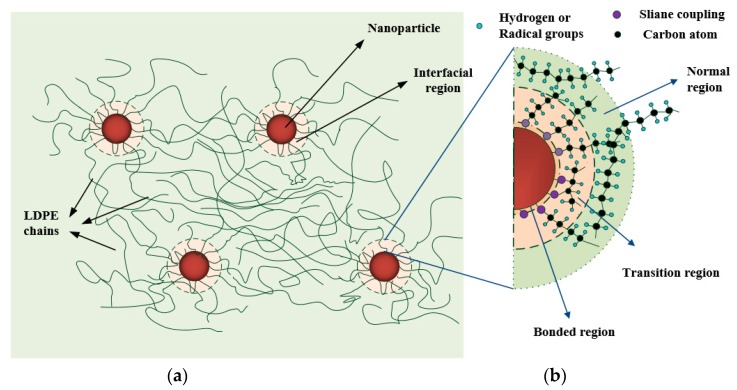
Interface region and morphology caused by surface modification of nanoparticles in LDPE/TiO_2_ nanocomposites. (**a**) Distributed nanoparticles with interface in LDPE matrix; (**b**) morphology in the interface region due to physicochemical interactions.

The introduced traps originated from the chemical and physical characteristics of the interface in the nanocomposites. Chemical surface modification of the nanoparticles can further enhance the breakdown performance by improving the nanoparticle–matrix coupling effects. Figure 9 shows the interface region and its morphology due to the surface modification of the coupling agent. According to the material preparation, surface treatment and the FTIR results, some polar groups and strong chemical bonds were introduced by the silane surface functionalization. The interface region around the nanoparticle surface was enhanced by surface modification. Some layers occurred in the interface region (has finite thickness). These layers were used for the stress transfer between the nanoparticles and the matrix and contributed to the enhancement of the overall free volume and the conformation of molecular chain segments [13]. Based on the potential barrier model [16], the KH560 molecules linked to the TiO_2_ nanoparticle by chemical bonds. The strong chemical bonds with the LDPE chain improved the movements of the chain segments in the transition region, resulting in the reorientation of the molecular chains, as shown in Figure 9. Outside the transition region, the LDPE chains presented a flexible structure, which was similar to the amorphous LDPE. The strong bonding effects on the nanoparticle surface by the polar groups in silane coupling yielded deep traps. In addition, the modified LDPE chains by the interactions in the transition region gave rise to the shallow traps. Above all, the surface modification of TiO_2_ nanoparticles responded to the improvements of interface morphology, contributing to the variation of trap parameters in the nanocomposites.

**Figure 10:**
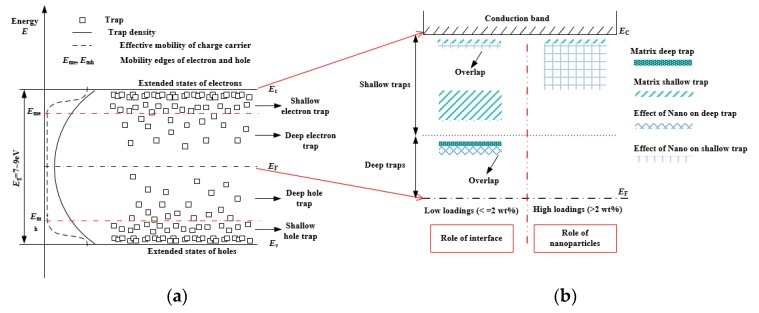
Trap variation due to interface region and nanoparticles in LDPE/TiO_2_ nanocomposites based on the energy band diagram. (**a**) Energy band structure; (**b**) effects of interface and nanoparticles on traps.

In order to understand the origin of the traps associated with the interface region, the “energy band structure” was employed here, which has been discussed for polyethylene dielectric in many papers [34,35]. Figure 10 shows the typical energy level structure and the state density of the polyethylene polymer. Shallow and deep traps refer to the localized states related to physical and chemical disorder, respectively [35]. The position of extended states of electrons refers to the conduction band minimum (*E*_c_), and the position of extended states of holes refers to the valence band maximum (*E*_v_). Deep traps were close to the Fermi level (*E*_F_) with a low density, while, shallow traps existed near the edge of the extended states, as shown in Figure 10a. In the low loading samples (≤2 wt%), the introduced new traps by the interface interacted with the original traps. On one hand, the new deep traps were formed. On the other hand, the original deep traps were modified by the overlap with the new deep traps, as shown in Figure 10b. Small amounts of shallow traps were introduced by the overlap effects, and the primary shallow trap density (*β* relaxation, 0.5~0.6 eV) was reduced. In the high loading samples (5 wt%, 10 wt%), the effects of TiO_2_ nanoparticles contributed to the significant increase of shallow traps (*γ* relaxation, 0.1~0.3 eV) by overlap effects. 

## 5. Conclusions 

Dielectric breakdown performance in LDPE/TiO_2_ nanocomposites was studied in this paper. Through tailoring the interface by surface modification of TiO_2_ nanoparticles, the dielectric response, trap parameters were improved. It indicated that the strength and the data dispersion of the breakdown were improved by TiO_2_ nanoparticles (<2 wt%).

Surface modification by KH560 silane coupling agent introduced some polar groups and chemical bonding on the surface of TiO_2_ nanoparticles. Consequently, the introduced new deep traps by slight TiO_2_ nanoparticles (<2 wt%) were detected in the nanocomposites. However, shallow traps significantly increased at high loadings (>2 wt%). It turned out that the large amounts of TiO_2_ nanoparticles contributed to the shallow traps.

The introduced deep traps by the small amount of TiO_2_ nanoparticles improved the breakdown properties of the LDPE. The strong bonding effects on the nanoparticle surface by silane coupling yielded deep traps. The original deep traps were modified by the overlap with the new deep traps. The improvement of the dielectric breakdown of polyethylene dielectric depended on the nanofiller loading. Particularly, the surface modification of nanoparticles could promote breakdown improvements in nanocomposites.

## Figures and Tables

**Table 1:** Trap parameters in LDPE/TiO_2_ nanocomposites.

Samples	Peak 1			Peak 2			Peak 3			Peak 4			Peak 5		
	*T*_m_ (°C)	*E*_t_ (eV)	*Q* (nC)	*T*_m_ (°C)	*E*_t_ (eV)	*Q* (nC)	*T*_m_ (°C)	*E*_t_ (eV)	*Q* (nC)	*T*_m_ (°C)	*E*_t_ (eV)	*Q* (nC)	*T*_m_ (°C)	*E*_t_ (eV)	*Q* (nC)
LDPE	/	/	/	76.5	1.10	1.05	50.9	0.61	6.60	/	/	/	−45	0.06	0.14
0.1 wt%	84.1	1.23	0.14	74.5	0.90	0.51	55.2	0.52	1.16	10.7	0.1	0.26	−43	0.13	0.23
0.5 wt%	84.5	1.42	0.43	73.6	0.91	0.45	58.1	0.65	0.78	7.5	0.1	0.18	−37	0.14	0.45
1 wt%	85.1	1.65	0.59	73.9	0.87	1.74	54.5	0.51	1.27	/	/	/	−23	0.15	0.62
2 wt%	84.2	1.45	1.15	72.6	0.72	1.1	/	/	/	/	/	/	−23	0.18	0.98
5 wt%	/	/	/	/	/	/	/	/	/	21.8	0.33	0.10	−33	0.2	2.82
10 wt%	/	/	/	/	/	/	/	/	/	/	/	/	−34	0.3	4.24

**Table 2:** Weibull distribution parameters of breakdown strength in LDPE/TiO_2_ nanocomposites.

Samples	*α*′ (kV/mm)	*β*′
Neat LDPE	223.28	4.33
0.1 wt%	238.97	4.84
0.5 wt%	262.77	7.86
1 wt%	265.55	6.36
2 wt%	229.61	3.89
5 wt%	210.95	3.10
10 wt%	202.27	5.38
